# Supplementary material for: TadA orthologs enable both cytosine and adenine editing of base editors
Source: Nat Commun. 2023 Jan 26;14:414. doi: 10.1038/s41467-023-36003-3 (PMC9880001; doi:10.1038/s41467-023-36003-3)
Supplement: Supplementary file 1 — Supplementary Information [file 41467_2023_36003_MOESM1_ESM.pdf]

# Supplementary Information
# TadA orthologs enable both cytosine and adenine editing of base editors

Shuqian Zhang et al.

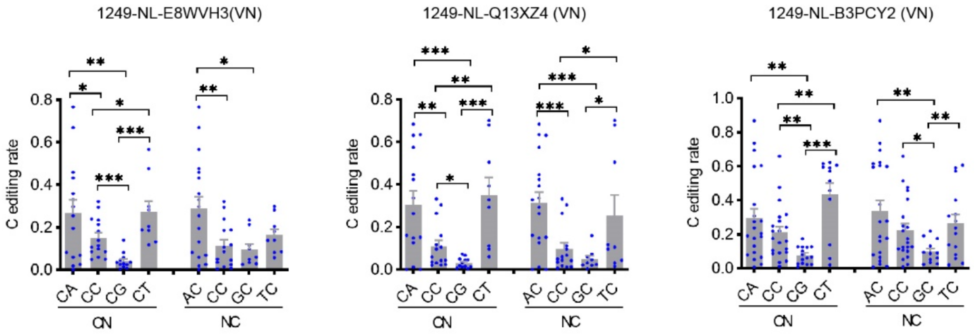


**Supplementary Figure 1. Sequence preferences of selected TadA ortholog-derived base editors.** Sequence preferences of selected TadA ortholog-derived base editors, including 49-NL-E8WVH3(VN), 49-NL-Q13XZ4(VN) and 49-NL-B3pcy2(VN). Data shown here represents means of results from n=3 biologically independent experiments. * represents $P$<0.05, ** represents $P$<0.01 and *** represents $P$<0.001 with one-tailed unpaired t-test for comparisons between every two groups. Exact $P$ values were provided in Source Data. Data are presented as mean values +/- SEM. Source data are provided as a Source Data file.

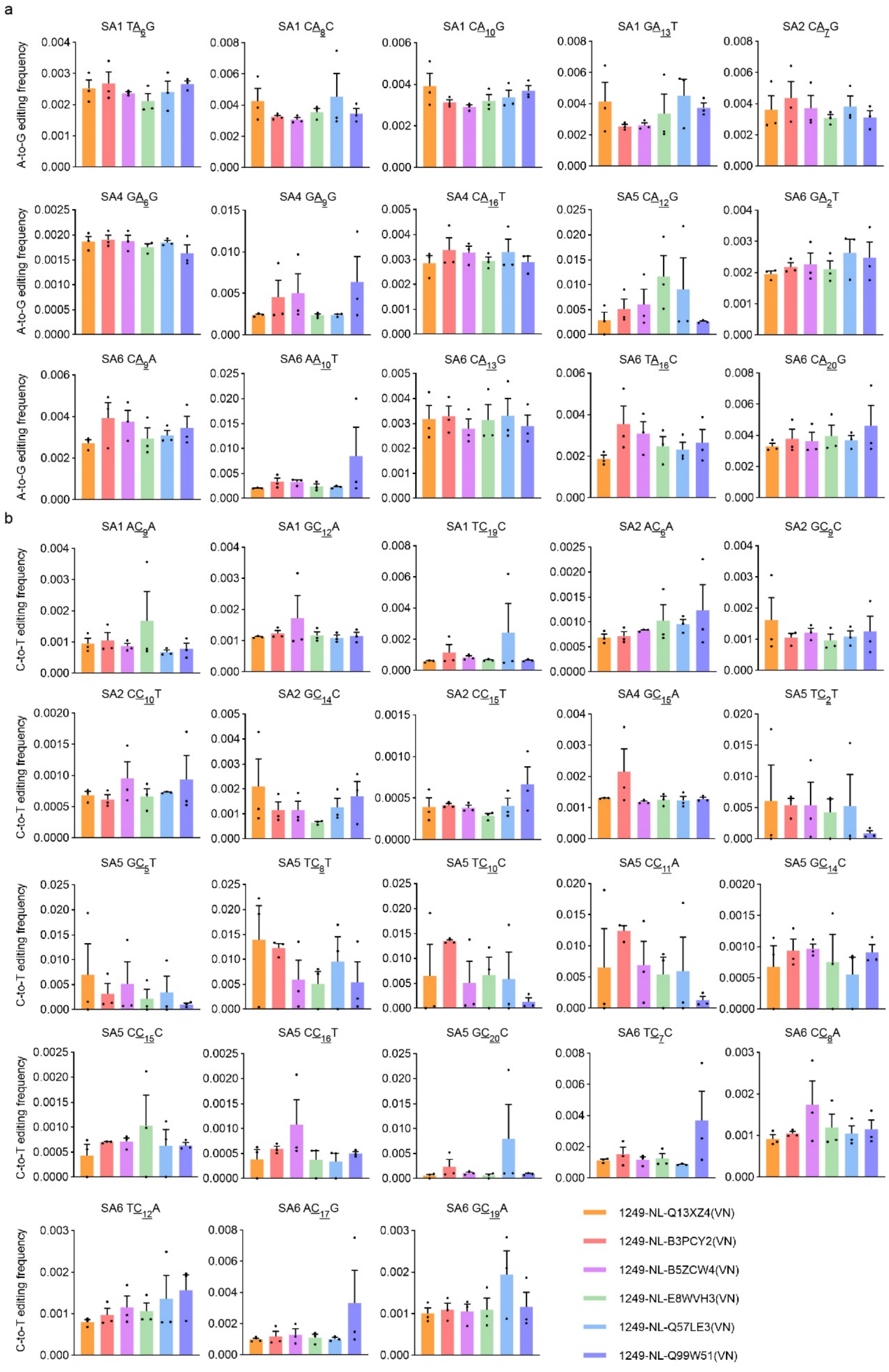


**Supplementary Figure 2. Cas9-independent DNA off-target edits of TadA ortholg-**

**derived base editors. a-b,** Cas9-independent DNA off-target edits of TadA ortholg-derived base editors were analyzed by R-loop assay and high-throughput targeted amplicon sequencing. A-to-G editing frequency (**a**) and C-to-T editing frequency (**b**) were all analyzed and quantified. Editing frequency for 1249-NL-Q13XZ4(VN), -B3PCY2(VN), -B5ZCW4(VN), -E8WVH3(VN), -Q57LE3(VN) and -Q99W51(VN) was depicted in orange, pink, purple, light green, blue and dark blue, respectively. Data shown here represents means of results from n=3 biologically independent experiments. Data are presented as mean values +/- SEM. Source data are provided as a Source Data file.

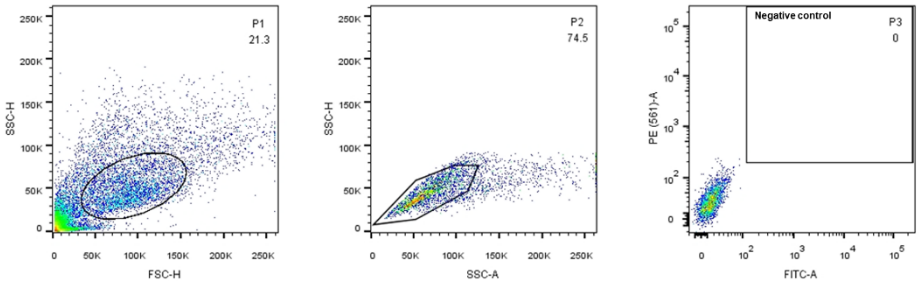


| Name ▲ | Statistic | #Cells |
| --- | --- | --- |
| NC-4.fcs | | 16368 |
| P1 | 21.3 | 3483 |
| **P2** | 74.5 | 2595 |
| P3 | 0 | 0 |

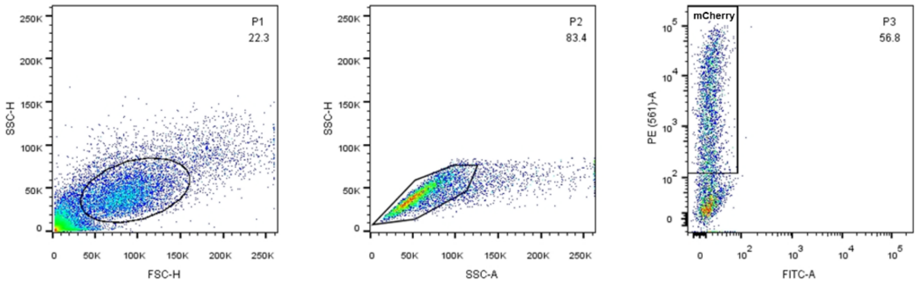


| Name ▲ | Statistic | #Cells |
| --- | --- | --- |
| mCherry-3.fcs | | 26910 |
| P1 | 22.3 | 6004 |
| **P2** | 83.4 | 5007 |
| P3 | 56.8 | 2846 |

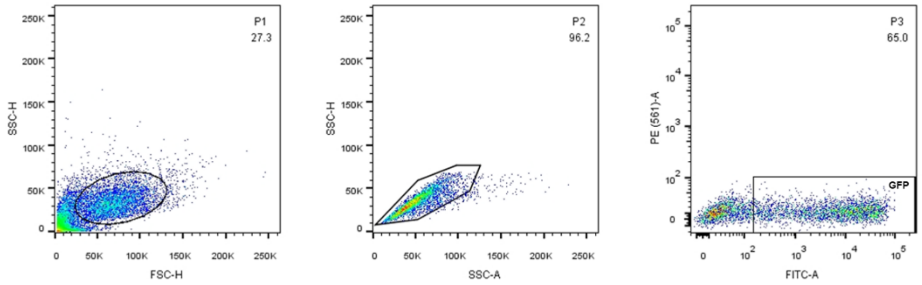


| Name ▲ | Statistic | #Cells |
| --- | --- | --- |
| GFP-7.fcs | | 18821 |
| P1 | 23.3 | 4391 |
| **P2** | 85.1 | 3735 |
| P3 | 50.1 | 1872 |

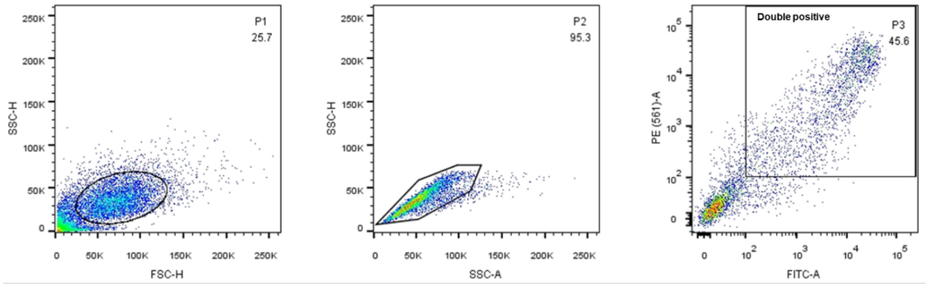


| Name ▲ | Statistic | #Cells |
| --- | --- | --- |
| GFP&mCherry-2.fcs | | 18538 |
| **P1** | 25.7 | 4761 |
| **P2** | 95.3 | 4536 |
| P3 | 45.6 | 2070 |

**Supplementary Figure 3. Representative FACS gating figures for Negative control,**

**mCherry, GFP and double positive samples.** Samples for on-target base editing activity analysis and R-loop assays were collected as double positive gating/sorting panels.
