# Supplementary material for: TadA orthologs enable both cytosine and adenine editing of base editors
Source: Nat Commun. 2023 Jan 26;14:414. doi: 10.1038/s41467-023-36003-3 (PMC9880001; doi:10.1038/s41467-023-36003-3)
Supplement: Supplementary file 2 — Reporting Summary [file 41467_2023_36003_MOESM2_ESM.pdf]

nature portfolio

Corresponding author(s): Tian-Lin Cheng

Last updated by author(s): Dec 13, 2022

Reporting Summary

Nature Portfolio wishes to improve the reproducibility of the work that we publish. This form provides structure for consistency and transparency in reporting. For further information on Nature Portfolio policies, see our Editorial Policies and the Editorial Policy Checklist.

Statistics

For all statistical analyses, confirm that the following items are present in the figure legend, table legend, main text, or Methods section.

n/a	Confirmed	
☐	☒	The exact sample size (*n*) for each experimental group/condition, given as a discrete number and unit of measurement
☐	☒	A statement on whether measurements were taken from distinct samples or whether the same sample was measured repeatedly
☐	☒	The statistical test(s) used AND whether they are one- or two-sided<br>*Only common tests should be described solely by name; describe more complex techniques in the Methods section.*
☒	☐	A description of all covariates tested
☐	☒	A description of any assumptions or corrections, such as tests of normality and adjustment for multiple comparisons
☐	☒	A full description of the statistical parameters including central tendency (e.g. means) or other basic estimates (e.g. regression coefficient) AND variation (e.g. standard deviation) or associated estimates of uncertainty (e.g. confidence intervals)
☐	☒	For null hypothesis testing, the test statistic (e.g. *F*, *t*, *r*) with confidence intervals, effect sizes, degrees of freedom and *P* value noted<br>*Give P values as exact values whenever suitable.*
☒	☐	For Bayesian analysis, information on the choice of priors and Markov chain Monte Carlo settings
☒	☐	For hierarchical and complex designs, identification of the appropriate level for tests and full reporting of outcomes
☒	☐	Estimates of effect sizes (e.g. Cohen's *d*, Pearson's *r*), indicating how they were calculated

Our web collection on statistics for biologists contains articles on many of the points above.

Software and code

Policy information about availability of computer code

Data collection	Next-sequencing data was collected with Illumina Novaseq. Double positive cells were collected with Flow cytometry (Moflo XDP, Beckman Coulter/ BD FACSAria™ Fusion Flow Cytometers). H2AX staining was analyzed with Fortessa Flow Cytometer (BD Biosciences),
Data analysis	For base editing analysis, CRISPResso2 was used.<br>For RNA-seq analysis, STAR (v2.4.2a), Sentieon genomics tools (v202010.02), Picard (v2.23.6, http://broadinstitute.github.io/picard), MuTect2 (202010.02),VariantFiltration tool (gatk-4.1.4.0).<br>H2AX staining was analyzed with Flowjo v10.

For manuscripts utilizing custom algorithms or software that are central to the research but not yet described in published literature, software must be made available to editors and reviewers. We strongly encourage code deposition in a community repository (e.g. GitHub). See the Nature Portfolio guidelines for submitting code & software for further information.

Data

Policy information about availability of data

All manuscripts must include a data availability statement. This statement should provide the following information, where applicable:

- Accession codes, unique identifiers, or web links for publicly available datasets
- A description of any restrictions on data availability
- For clinical datasets or third party data, please ensure that the statement adheres to our policy

The raw high-throughput sequencing data generated in this study have been deposited in the NCBI sequence Read Archive database under PRJNA758206 (https://www.ncbi.nlm.nih.gov/bioproject/PRJNA758206) and PRJNA757902 (https://www.ncbi.nlm.nih.gov/bioproject/PRJNA757902). GRCh38 reference genome GRCh38.v96 used in this study is available at https://ftp.ensembl.org/pub/release-96/gtf/homo_sapiens/Homo_sapiens.GRCh38.96.gtf.gz. All plasmids described in this work are available on reasonable request and would be deposited in Addgene. Source data are provided with this paper.

Human research participants

Policy information about studies involving human research participants and Sex and Gender in Research.

Reporting on sex and gender	N/A
Population characteristics	N/A
Recruitment	N/A
Ethics oversight	N/A

Note that full information on the approval of the study protocol must also be provided in the manuscript.

Field-specific reporting

Please select the one below that is the best fit for your research. If you are not sure, read the appropriate sections before making your selection.

[x] Life sciences [ ] Behavioural & social sciences [ ] Ecological, evolutionary & environmental sciences

For a reference copy of the document with all sections, see nature.com/documents/nr-reporting-summary-flat.pdf

Life sciences study design

All studies must disclose on these points even when the disclosure is negative.

Sample size	No statistical analysis were performed to predetermine sample size. but our sample sizes are in accordance to those generally used in this field. For orthologous TadA screening, 2 biological replicates were performed. For all other experiments performed in cell lines, at least 3 biological replicates were performed.
Data exclusions	No data was excluded.
Replication	Biological replicates and triplicates were performed independently. All attempts at replication were successful.
Randomization	Cells used in this study were grown under identical conditions, no randomization was used.
Blinding	Blinding was not used, because no subjective assessments were required.

Reporting for specific materials, systems and methods

We require information from authors about some types of materials, experimental systems and methods used in many studies. Here, indicate whether each material, system or method listed is relevant to your study. If you are not sure if a list item applies to your research, read the appropriate section before selecting a response.

Materials & experimental systems

n/a	Involved in the study
[ ]	[x] Antibodies
[ ]	[x] Eukaryotic cell lines
[x]	[ ] Palaeontology and archaeology
[x]	[ ] Animals and other organisms
[x]	[ ] Clinical data
[x]	[ ] Dual use research of concern

Methods

n/a	Involved in the study
[x]	[ ] ChIP-seq
[ ]	[x] Flow cytometry
[x]	[ ] MRI-based neuroimaging

Antibodies

Antibodies used: Alexa Fluor® 647 Mouse anti-H2AX (pS139) (BD Pharmigen) catalog 560447 (1:10 for flow cytometry)

Validation: Alexa Fluor® 647 Mouse anti-H2AX (pS139) : validated by manufacturer by immunofluorescence in Hela cells and by flow cytometry in human perihperal blood mononuclear cells(PBMC).

Eukaryotic cell lines

Policy information about cell lines and Sex and Gender in Research

Cell line source(s): HEK293T cells (GNHu17, Cell Bank of the Chinese Academy of Sciences, Shanghai, China)

Authentication: Cells were authenticated by the supplier using STR analysis.

Mycoplasma contamination: Cells tested negative mycoplasma.

Commonly misidentified lines (See ICLAC register): None commonly misidentified lines used.

Flow Cytometry

Plots

Confirm that:

- [x] The axis labels state the marker and fluorochrome used (e.g. CD4-FITC).
- [x] The axis scales are clearly visible. Include numbers along axes only for bottom left plot of group (a 'group' is an analysis of identical markers).
- [x] All plots are contour plots with outliers or pseudocolor plots.
- [x] A numerical value for number of cells or percentage (with statistics) is provided.

Methodology

Sample preparation: HEK293T cells were passaged and plated into 6-well or 48-well plates (Corning) for transfection. Base editor- and sgRNA-expressing plasmid (mole ratio 2:1) were mixed with 2.5 µl (48-well) or 8 µl (6-well) Lipo293TM (Beyotime biotechnology, Shanghai, China) for transient transfection. For base editing analysis, 72 hours after transfection, cells in 48-well plates were digested and prepared for flow cytometry to collect double positive cells.
For H2AX staining, cells were collected and washed with PBS, and fixed with 4% paraformaldehyde at room temperature (25° C) for 15 min. Following washes with PBS, cells were permeabilized with 0.5% Triton X-100 in PBS for 2 min. Next, block cells with buffer (10% goat serum in TBS) at room temperature (25°C) for 1 h. For the detection of DNA damage, stain the cells with γ-H2AX antibody (BD Pharmigen, 647) at 4℃ for 2 h and wash with PBS. Cells were gated on fluorescein isothiocyanate and allophycocyanin using the Fortessa Flow Cytometer (BD Biosciences), and results were analyzed by FlowJo v10.

Instrument: Moflo XDP, Beckman Coulter/ BD FACSAria™ Fusion Flow Cytometers/BD Fortessa Flow Cytometer

Software: FlowJo X 10.0.7

Cell population abundance: cell population abundances for target populations were similar across different base editor experiments. Transfected HEK293T cells usually were ~ 20-40% GFP/mCherry double positive.

Gating strategy: Gates were established using untransfected control cells, transfected GFP+ cells and transfected mCherry+ cells. Gates were drawn to collect all double positive cells.

- [x] Tick this box to confirm that a figure exemplifying the gating strategy is provided in the Supplementary Information.
