# Supplementary material for: TadA orthologs enable both cytosine and adenine editing of base editors
Source: Nat Commun. 2023 Jan 26;14:414. doi: 10.1038/s41467-023-36003-3 (PMC9880001; doi:10.1038/s41467-023-36003-3)
Supplement: Supplementary file 3 — Description of Additional Supplementary Files [file 41467_2023_36003_MOESM3_ESM.pdf]

Title: Supplementary Data 1
Description: Detailed protein sequence information for 1000 TadA orthologs

Title: Supplementary Data 2
Description: Detailed protein sequence information for 54 selected TadA orthologs used for functional screening

Title: Supplementary Data 3
Description: Editing activities at five endogenous sites of 54 TadA ortholog-derived base editors

Title: Supplementary Data 4
Description: Information of sgRNAs used in this study
